# Rare-Earth Free Self-Activated Graphene Quantum Dots and Copper-Cysteamine Phosphors for Enhanced White Light-Emitting-Diodes under Single Excitation

**DOI:** 10.1038/s41598-017-13404-1

**Published:** 2017-10-09

**Authors:** Wubin Dai, Yifeng Lei, Man Xu, Pei Zhao, Zhanhui Zhang, Jia Zhou

**Affiliations:** 1 Key Laboratory for Green Chemical Process of Ministry of Education, Hubei Key Laboratory of Plasma Chemistry and Advanced Materials, School of Material Science and Engineering, Wuhan Institute of Technology, Wuhan, 430205 Hubei China; 2 The Institute of Technological Science, School of Power and Mechanical Engineering, Wuhan University, Wuhan, 430072 Hubei China

## Abstract

Rare-earth (RE) based phosphors are attractive due to their potential applications. However, owing to the resource issue, these kinds of phosphors are expensive and costly. On the contrary, as for phosphor-convert white light-emitting-diodes (pc-WLEDs), a solution-processed tunable warm white emission LED composite is fabricated in this study under single excitation, with both RE free phosphors graphene quantum dots (GQDs) and Copper-Cysteamine (Cu-Cy). By using microwave-assisted wet-chemical method and with graphite as raw material, cold white fluorescence of the GQDs is obtained. Cu-Cy which shows intense photoluminescence in the red region has the structure where both the thio and amine groups connected with copper and forming cysteamine. Warm white light is achieved by mixing the two self-activated RE free phosphors at the weight ratio of 1: 1.7 under the excitation at 365 nm. The designed optimal LED device has the properties of CIE (x, y) = (0.341, 0.327), T = 4436 K, R = 87.9 EQE = 0.31%. The experimental results demonstrate that RE free phosphor(s) excited under a single chip can open up a new avenue to develop much lower device for warm WLEDs.

## Introduction

Luminescence is defined as the phenomenon in which absorbed energy of a luminescent material excited by external energy is given off as photons, resulting in the form of different light emission, and which has been playing a major scientific and technological role in different fields of science^1–3^. With a significant portion of global energy consumption going toward lighting, it is important to develop efficient, illumination-grade lighting technologies. Since white light emitting diodes (WLEDs) came into commercial use in 1997, they have been in increasing demand as a potential replacement for conventional light sources due to their advantages in low power consumption, high luminous efficiency, and long lifetime^4^. Driven by the development of WLEDs, a variety of luminescent materials also called phosphors, which are composed of activators and/or sensitizers (generally are rate-earth (RE) and/or transition metal ions) with a suitable matrix, have rapidly emerged and their luminescence properties have also been improved following time. Actually, RE phosphors have not only advantages in fabricating WLEDs but also have many potential applications in other techniques, such as solar cells, biomedical sensing^5–7^. However, these phosphors also cause the inevitable problems that RE elements are very expensive and their resources in the crust are very limited due to the extremely low abundance. To solve this problem, RE free phosphors are the candidates needed to explore and should meet the following two conditions: 1) the optical properties are comparable to the RE phosphors to fulfill the practical applications and 2), low synthetic price and/or easy to obtain. This concept represents a new research direction for luminescence and light converting phosphors.

Due to their high fluorescent quantum yield, narrow emission bandwidth and resistance to the photo-bleaching, the potential of using semiconductor nanocrystals, especially semiconductor quantum dots in photonics and optoelectronics has been realized by recent progresses in developing various devices including optical modulator, LEDs, photo-catalysis, ultrafast lasers and photodetectors^8–10^. Some studies show that integration of quantum dots (QDs) into WLEDs can overcome inherent problems such as low environmental stability and device lifespan. However, the overall (external) quantum efficiency (EQE) of the QDs-WLEDs is still very low, such as the EQE of 0.0013% for the CdSe-WLEDs^11^. Moreover, semiconductor QDs typically contains heavy toxic metals, which limits its further research and development^12,13^. Instead, graphene QDs (GQDs) is classified as carbon dots, which is fabricated by graphite and have better properties compared to semiconductor QDs due to the low toxicity, high carrier transport mobility and stable photoluminescence^14,15^. The GQDs are considered as novel material for biological, optoelectronics, energy and environmental applications. As application in WLEDs, high surface activity makes GQDs could graft other functional groups (polymers and organic molecules), which leads the GQDs with dispersion property in some organic solvent and suitable for constructing LED devices. Some previous reports have achieved the use of GQDs in the WLEDs^16,17^. However, the luminance, EQE, color-rendering index (CRI) and correlated color temperature (CCT) are all not meeting the requirements for practical application. Therefore, to develop novel GQDs-WLEDs with enhanced properties is still urgent work.

Meanwhile, due to the lack of suitable red light in the emission, it is difficult to make high quality of warm WLEDs with the performances of high CRI and low CCT. Both of these two properties are key factors for some important applications such as indoor lighting. These drawbacks can be completely overcome by mixing a red phosphor in LEDs to complement the red light component^18,19^. To this regard, great effects have been made to exploit different red phosphors, which the most used are the RE phosphors and successful materials are Eu^2+^ and/or Ce^3+^-activated (oxy)nitride compounds^20,21^. As mentioned, these kinds of phosphors are costly and not conducive to large-scale application. Instead, the RE free metal complexes, which have potential applications as phosphor in different fields, are the last few years to become a research hotspot due to they can be obtained simply *via* a green chemistry synthesis without harm to the environment and have adjustable luminous performance. Among different ligands, cysteamine (HSCH_2_CH_2_NH_2_), which consists of small organic molecule with both active thiol and amino groups, performs strong metal affinity (i.e., Cu) and functions. Various Cu-cysteamine (Cu-Cy) complexes have been studied and different crystal structures have different properties^22–24^. Herein, we report a Cu-Cy complex Cu_3_Cl(SR)_2_ (R=CH_2_CH_2_NH_2_), which has the luminescence in the red region under 365 nm excitation and suitable for the red component to produce warm WLEDs.

Moreover, we further designed a composition of warm WLEDs, which contains the RE free GQDs and Cu-Cy phosphors under a single wavelength excitation (365 nm). The optimal warm WLEDs show that the white light emission with an external quantum yield of 0.31% can be achieved by mixing these two self-activated luminescent materials at the weight ratio of 1: 1.7. Using the near-ultraviolet (NUV) chip for excitation, the composition of the LEDs emits white light that exhibits an excellent CRI (87.9) and CCT (4436 K). These materials are environment friendly, easy to synthesize, and cost-effective. Meanwhile, a single chip and free phosphors used in this study can greatly reduce the cost of the device.

## Results

### Structure and optical properties of GQDs

**Figure 1:**
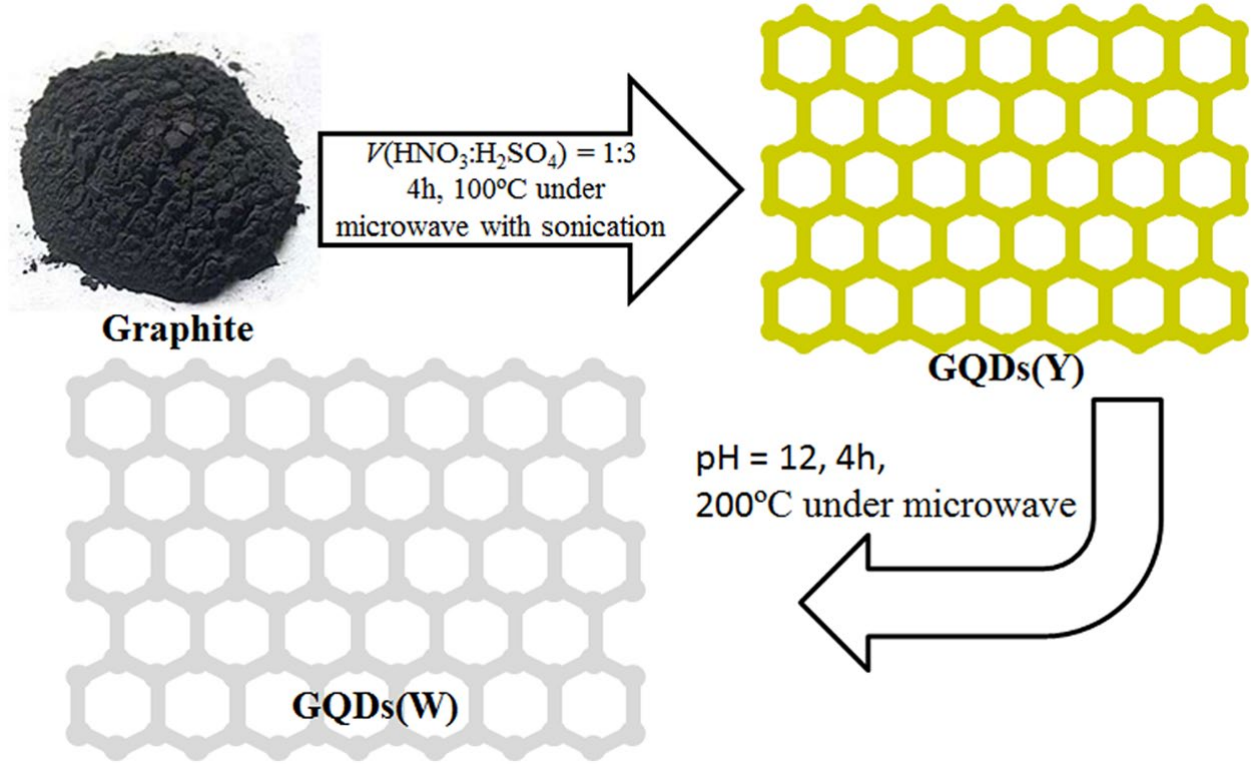
Preparation processes of the GQDs(Y) and GQDs(W).

**Figure 2:**
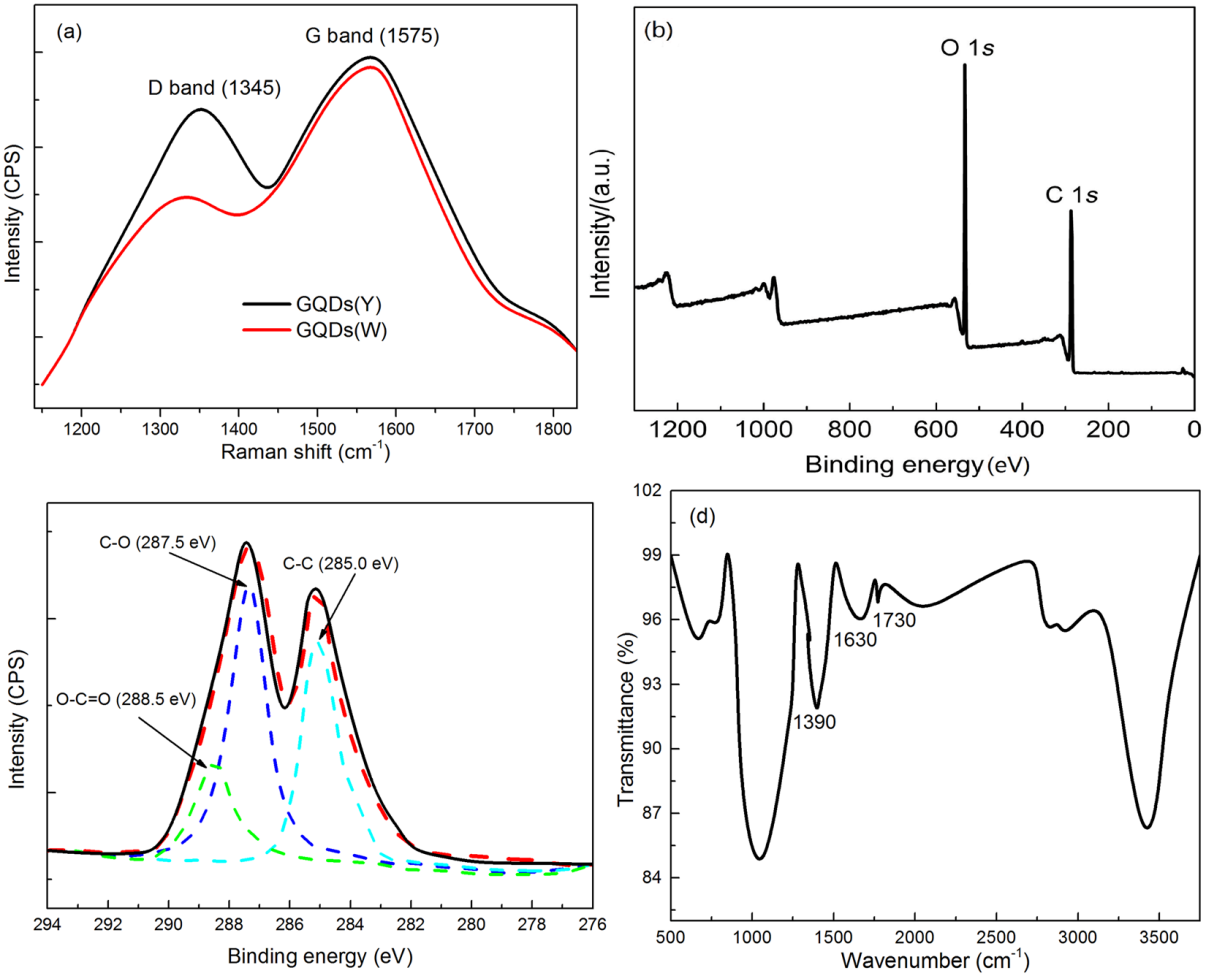
(**a**) Raman spectra of GQDs(Y) and GQDs(W), (**b**) Full-scan XPS spectrum of GQDs(W), (**c**) High-resolution C1s XPS spectrum of GQDs(W), (**d**) FTIR spectrum of GQDs(W).

The GQDs was obtained *via* the facile two-step microwave-assisted synthesis process (Fig. 1). Yellow fluorescent GQDs(Y) were firstly obtained through exfoliation of the oxidized graphite with the assistant of sonication and microwave irradiation (100 °C). Then, the solution of the sample was further reacted through microwave (200 °C) under alkaline condition (pH = 12), which results in GQDs(W) with cold white emission. The GQDs(W) was then adopted as the raw material to produce WLED device by the solution-processing method. Raman spectra (Fig. 2a) of GQDs show peaks located at ~1345 and 1575 cm^−1^, which are the D and G bands of graphene, respectively^25,26^. Compared with the intensities of D and G bands for GQDs(Y) and GQDs(W), the ratios (*I*_G_/*I*_D_) are 1.12 (GQDs(Y)) and 1.45 (GQDs(W)), respectively, which indicate that the decrease of defects in the structure. The binding energy peaks located at 284.8 and 536 eV (Fig. 2b) are corresponding to C1s and O1s, respectively, which imply that the GQDs(W) contains the element of O and C^27^. Meanwhile, the deconvolution of the C1s XPS spectrum (Fig. 2c) shows the peaks of binding energies at ~285.0, 287.5 and 288.5 eV, which are corresponding to the atomic valence bonds of CC, C-O and O-C=O, respectively^27,28^, and indicating the surface oxidation of graphene during the preparation. For comparison, the binding energy peaks and the deconvolution of the C1s XPS spectra were also performed for the GQDs(Y) (Supplementary Fig. S1). In addition to changes in intensities, the spectra positions have not changed at all because of further hydrothermally reduced under high temperature reaction and decreasing of defects in the GQDs(W) compared with GQDs(Y). The absorption bands (FTIR spectrum, Fig. 2d) are located at ~1390, 1630 and 1730 cm^−1^, which can be recognized to C=O vibration in carboxyl group, aromatic stretching vibration (graphitic domains) and the O-H (originated from C-OH group), respectively^14,29^. Due to the functional groups and the high specific surface energy, the GQDs are suitable for fabrication of LEDs device *via* solution chemical process.

**Figure 3:**
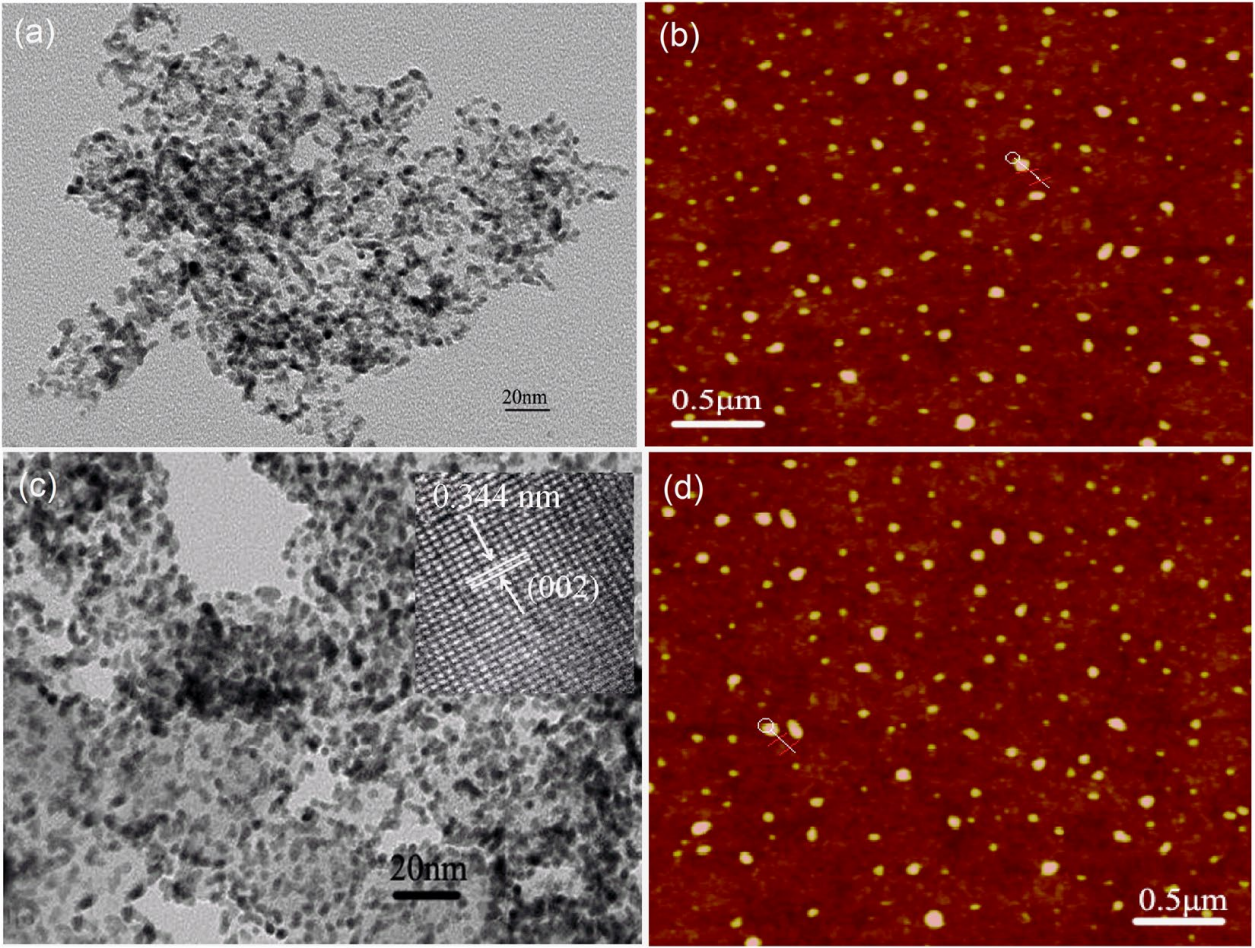
TEM images of the GQDs(Y) (**a**) and GQDs(W) (**c**), AFM images of the GQDs(Y) (**b**) and GQDs(W) (**d**).

The morphology of the GQDs is also observed using the TEM and AFM. Figure 3a shows the TEM image for the GQDs(Y) exhibits that the average lateral size is ~5 nm (the particle size distribution is shown in Supplementary Fig. S2a). Figure 3b shows the AFM height image which is calculated to be ~4 nm (height distribution is shown in Supplementary Fig.S2b). Meanwhile, the lateral size of GQDs(W) is a little bigger (6–9 nm) while the height is smaller (1.2–2.6 nm), which indicate that the finally obtained GQDs(W) are consisted of multilayer structure (Fig. 3c,d and Supplementary Fig. 2c,d). Moreover, the crystal lattice spacing is ~0.344 nm (inset of Fig. 3c), which can be specified as the (002) plane of graphite.

**Figure 4:**
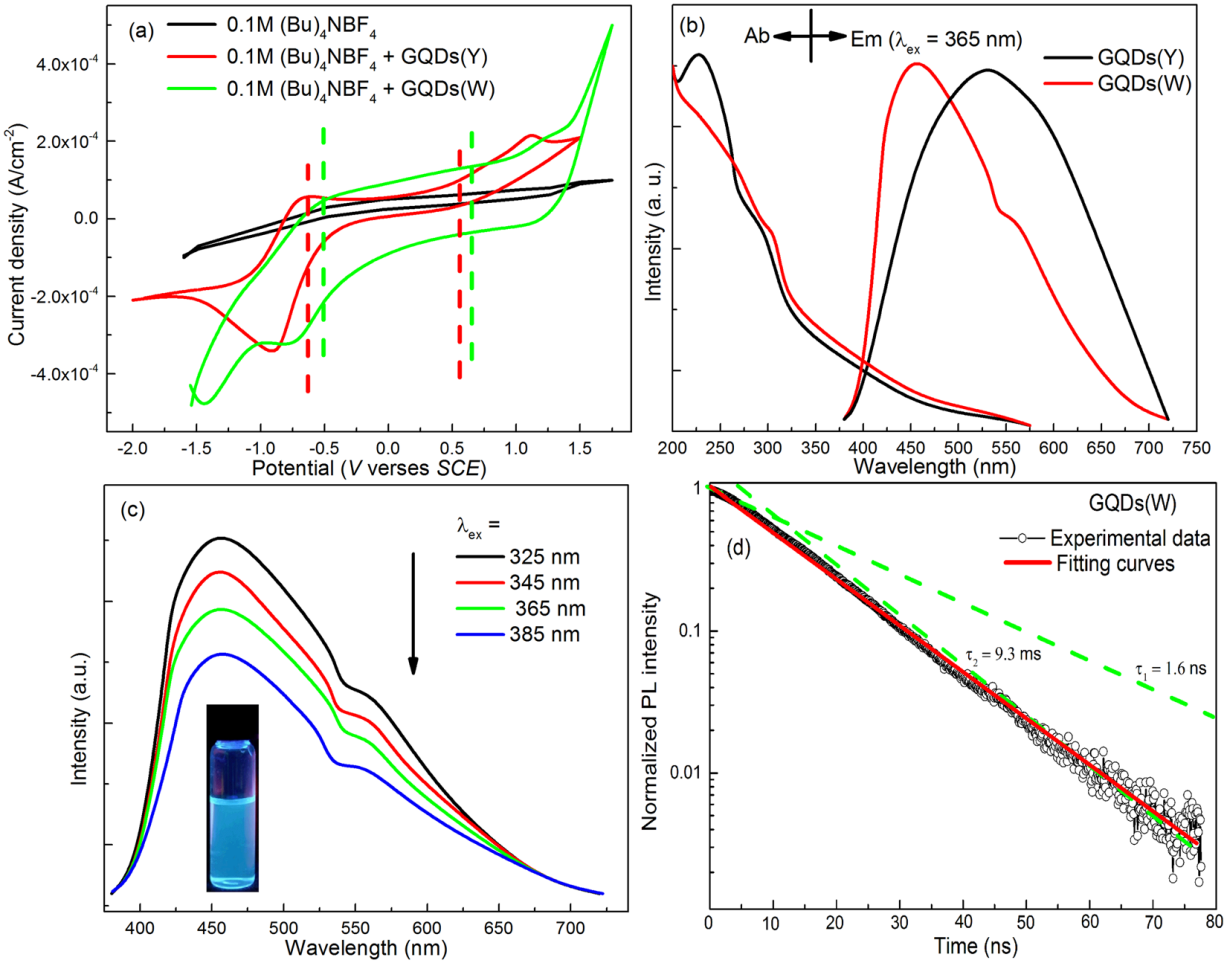
(**a**) *CV* spectra at a scan rate of 100 mV·s^−1^, (**b**) Absorption and emission spectra of GQDs(Y) and GQDs(W), (**c**) PL spectra of GQDs(W) following different excitation wavelength, the inset shows the fluorescent image of GQDs(W) aqueous solution at λ_ex_ = 365 nm, (**d**) fluorescent lifetime of GQDs(W) at λ_ex_ = 365 nm.

To investigate the optical properties, the band gap of the GQDs was studied. For estimating the highest occupied molecular orbital (HOMO) and lowest unoccupied molecular orbital (LUMO) energy levels, cyclic voltammetry (*CV*) was performed *via* establishing a standard three electrode system, which is composed of the working electrode (glassy carbon electrode), the counter electrode (platinum wire) and the reference electrode (saturated calomel electrode). *CV* was conducted at the scan rate of 0.15 V·s^−1^ in the DMF containing GQDs and 0.2 M (Bu)_4_NBF_4_ as the supporting electrolyte. The energy levels of GQDs were estimated by the empirical formulas^17,30^: E_(HOMO)_ = −(E_ox_ + 4.74) (eV) and E_(LUMO)_ = −(E_red_ + 4.74) (eV), where E_ox_ and E_red_ are the onset of oxidation and reduction potential, respectively. The curves in the *CV* were shown in the Fig. 4a, which can be obtained that the E_ox_ = 0.71 and 0.56 eV and E_red_ = −0.44 and −0.65 eV (for the GQDs(W) and GQDs(Y)), respectively. Hence, the HOMO and LUMO of the GQDs(W) and GQDs(Y) were calculated to be ~5.45 and 4.30, 5.30 and 4.09 eV, respectively. Therefore, the band gaps were ~1.15 eV (GQDs(W)) and 1.21 eV (GQDs(Y)), respectively, which indicates that the band gap of the GQDs(Y) is wider than that of GQDs(W).

The normalized PLE and PL spectra of the GQDs were shown in the Fig. 4b. For the GQDs(Y), two absorption bands at ~230 and 265 nm can be ascribed to the π-π* transition of *sp* ^2^ domain in GQDs, while the weaker shoulder band at ~315 nm can be assigned to the *n*- π* transition of oxygen-containing functional group (C=O)^31^. Meanwhile, the missing of the 230 nm PLE band for the GQDs(W) is due to the further hydrothermally reduced under high temperature reaction. The PL spectra of one emission band at ~523 nm can be observed for the GQDs(Y) under λ_ex_ = 365 nm while there are bands in the PL spectra for the GQDs(W) at the ~450 nm along with a weak peak at ~560 nm (the CIE coordinates of the samples were shown in the Supplementary Fig. S3). This could be associated with the further hydrothermally reduced under the second microwave radiation. The cold-white fluorescence of the GQDs(W) aqueous solution can be clearly observed (inset of Fig. 4c, under λ_ex_ = 365 nm). The emission intensities of GQDs(W) were decreasing with the varieties of excitation wavelength from 300 to 400 nm (Fig. 4c). The decay lifetime of GQDs was measured by the time-resolved fluorescence decay with single photon counting (Fig. 4d), which shows that there are two lifetime components with the amplitude of 6.9% (9.3 ns) and 93.1% (1.6 ns), respectively. The results indicate that there are two different luminous centers and this short lifetime made the GQDs(W) suitable for preparation the LEDs device. The two different luminous centers indicate that the GQDs have distinct chemical structures which possess different energy gap (~0.4 eV) between the singlet ground state (S_0_) and the first-excited state (S_1_). Actually, the similar double-peak PL phenomena could be regularly observed in polyaromatic systems, which is associated with the C-C inter-ring or intra-ring stretch modes^32,33^.

### Structure and optical properties of Cu-Cy

**Figure 5:**
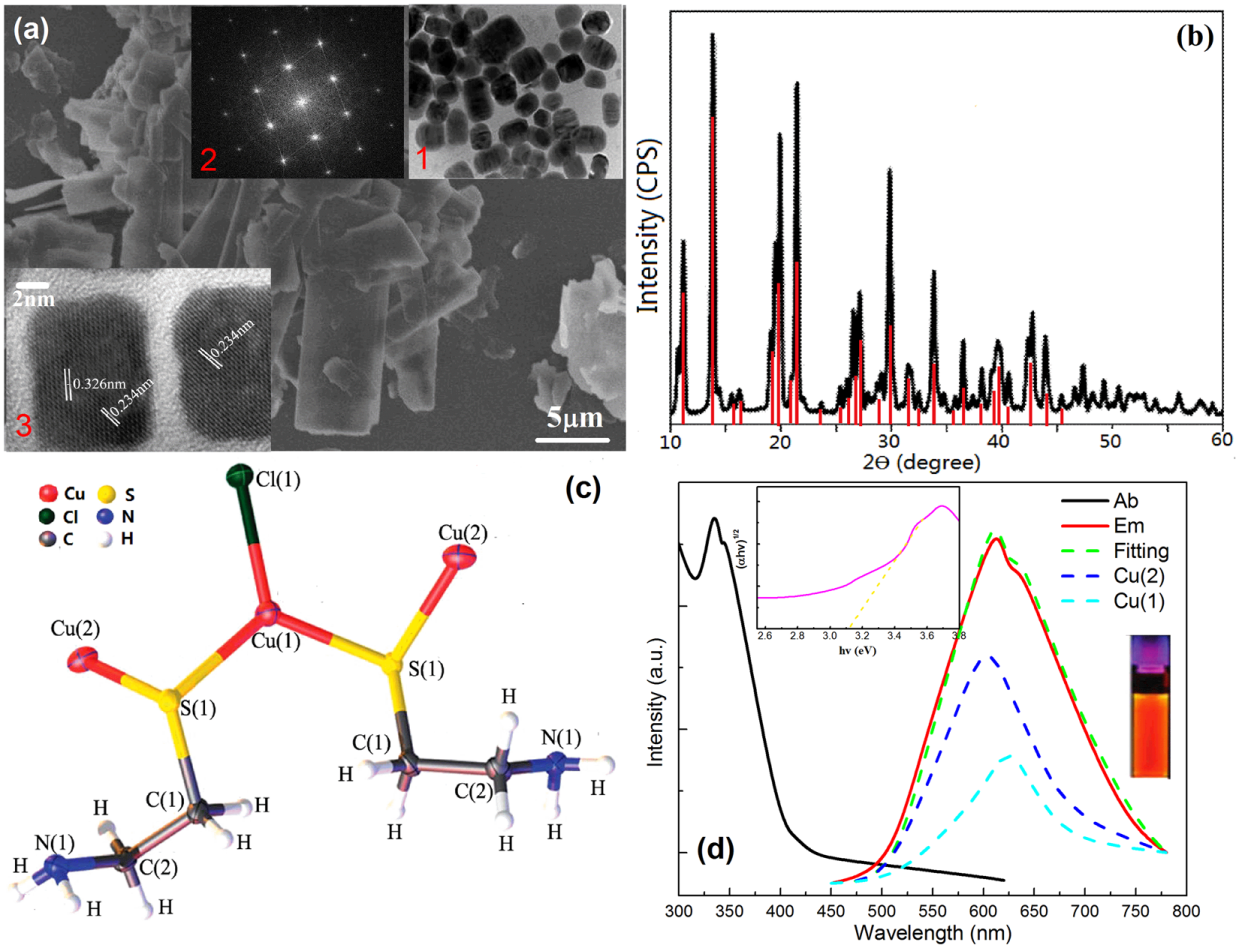
(**a**) SEM image of Cu-Cy, the insets 1 and 3 are HRTEM images, 2 is electron diffraction pattern, (**b**) powder XRD pattern, (**c**) crystal structure and (**d**), absorption and emission spectra, the insets show the calculated band gap and the digital picture of the Cu-Cy (under λ_ex_ = 365 nm).

Cu-Cy (Cu_3_Cl(SR)_2_, R=CH_2_CH_2_NH_2_) is a material with structure where both thio and amine groups form cysteamine and bonding with Cu^22–24^. This material can be synthesized simply *via* wet chemical method with no harm to the environment due to cysteamine is nontoxic and soluble in water. The morphology observed from SEM (Fig. 5a) of the powder Cu-Cy is regular shaped as sheet rectangular with the average size ranges ~ length (5–10 μm) × width (3–5 μm). Together with the larger-sized crystals, some smaller size (in the 10–100 nanometers scale) crystals could also be observed (Fig. 5a -1 (HRTEM)). The electron diffraction pattern (Fig. 5a -2) indicates that these small crystals are single crystals. Meanwhile, the lattice spaces measured from the HRTEM are *d*_1_ = 0.326 nm and *d*_2_ = 0.234 nm, respectively (Fig. 5a -3). The XRD patterns (Fig. 5b) of the powder shows very sharp and intense peaks, which means that the particles are relatively large and highly crystalline, and consistent with the observations from the SEM and TEM. The lattice spacing calculated from the XRD lines at 25.50° and 40.10° are ~0.318 nm and 0.230 nm, respectively, which are in agreement with the HRTEM measurements. Moreover, the particle size calculated by the Scherrer Equation: *d* = *Kλ/βcosθ*, where *d* is the mean size of the crystalline domains, *K* is a dimensionless shape factor (with a value close to unity), *λ* is the X-ray wavelength, *β* is the line broadening at FWHM and *θ* is the Bragg angle, respectively. From the XRD peaks at the two most intense indexed peaks 13.6° and 21.7 °, the *d*-values are calculated to be ~62.5 nm and 54.7 nm, respectively, which are in agreement with the HRTEM observations that some smaller size crystals exist in the structure. These results confirm the obtained Cu-Cy material is the Cu_3_Cl(SR)_2_.

**Figure 6:**
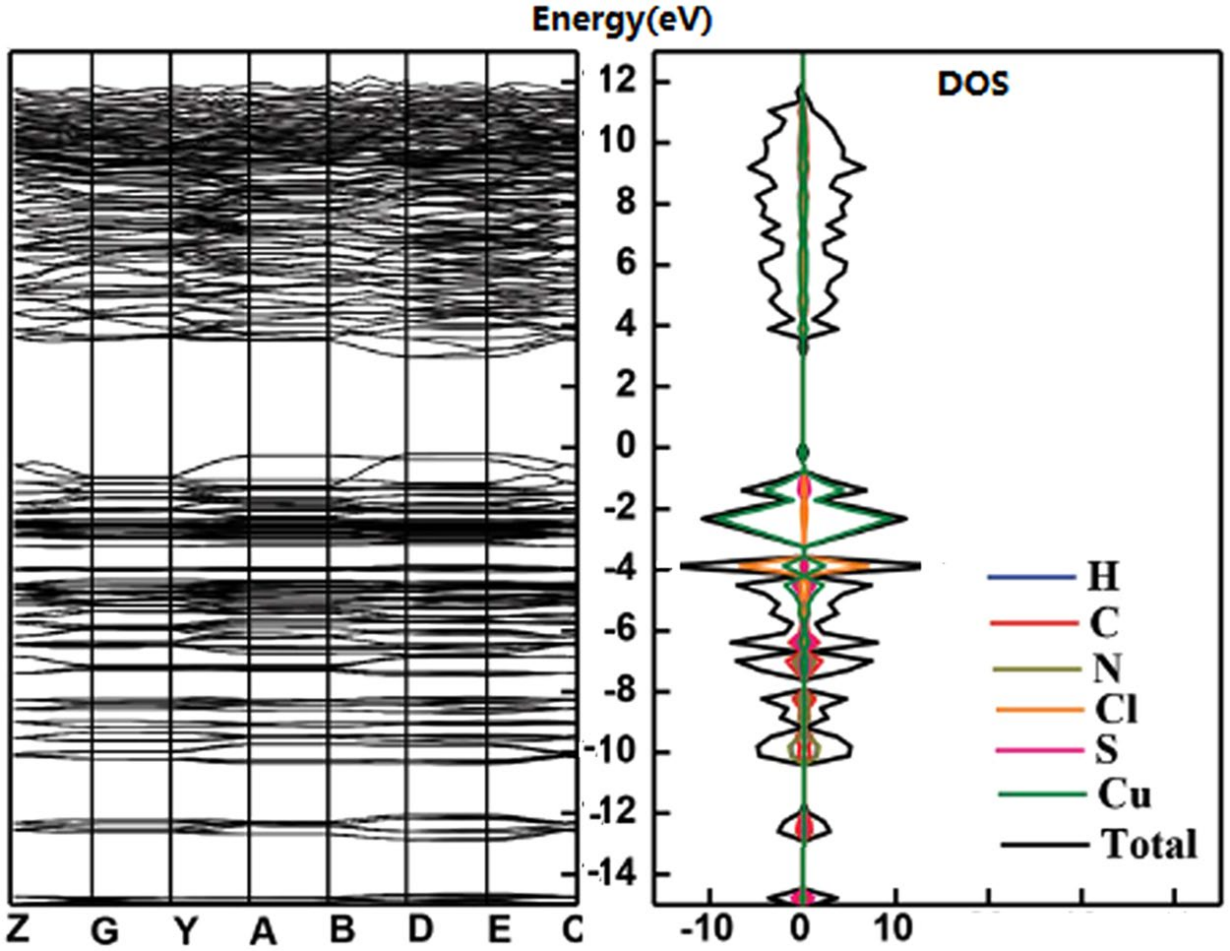
Left, band structure and right, DOS calculated *via* hybrid DFT.

The Cu-Cy has two different sites for Cu atoms and the solid-state nuclear magnetic spectroscopy and valence analyses from single crystal XRD have shown that they are both +1 valence^34,35^. The Cu(1) is coordinated with two S ions with average distance of 2.233 Å and one Cl atom with distance of 2.293 Å. The Cu(2) is connected with two S atoms with average distance of 2.302 Å and one N with distance of 2.091 Å (Fig. 5c). Based on the First-principle, the band structure and DOS of Cu-Cy calculated are shown in the Fig. 6, which calculated the band gap (E_indirect_) is ~3.14 eV. Meanwhile, the Cu 3d state are in the between of conduction band minimum (CBM) and valence band maximum (VBM). Under UV excitation, electrons would be excited from VB to CB upper level (absorption spectra) due to the transition between the VBM and CBM is forbidden according to the dipole and spin selection rules. The absorption and emission spectra of Cu-Cy are shown in Fig. 5d. The absorption spectrum show an edge at ~398 nm and the band gap estimation is shown in the inset of Fig. 5d based on the Kubelka-Munk function. The measured value of the band gap is ~3.10 eV, which is close to the DFT calculation result. The emission spectrum is broadband with main peaks located at ~615 nm and 630 nm under λ_ex_ = 365 nm. The large Stoke shift cannot be designed to the charge transfer transition from the ligand to Cu 3d state, instead, these emission bands are originated from the Cu 3d^9^4s^1^-3d^10^ transition and the ligand empty π* orbit to Cu 3d^10^ transition. For Cu1, the connected anion cannot give empty orbits for electron transition. Therefore, the longer wavelength emission (630 nm) can be ascribed to the 3d^9^4s^1^-3d^10^ transition (Cu1). For Cu2, the coordination N ion forms the sp^3^ hybridization with the nonbonding orbits and long pair electrons, thus, transition between π*-3d^10^ results in the short wavelength emission (615 nm). It is interesting and important that the GQDs and Cu-Cy have similar excitation spectra (both have broad excitation band in the range from 250 to 420 nm), which indicates that the two phosphors can be activated *via* a single wavelength excitation.

### Performances of WLEDs

**Figure 7:**
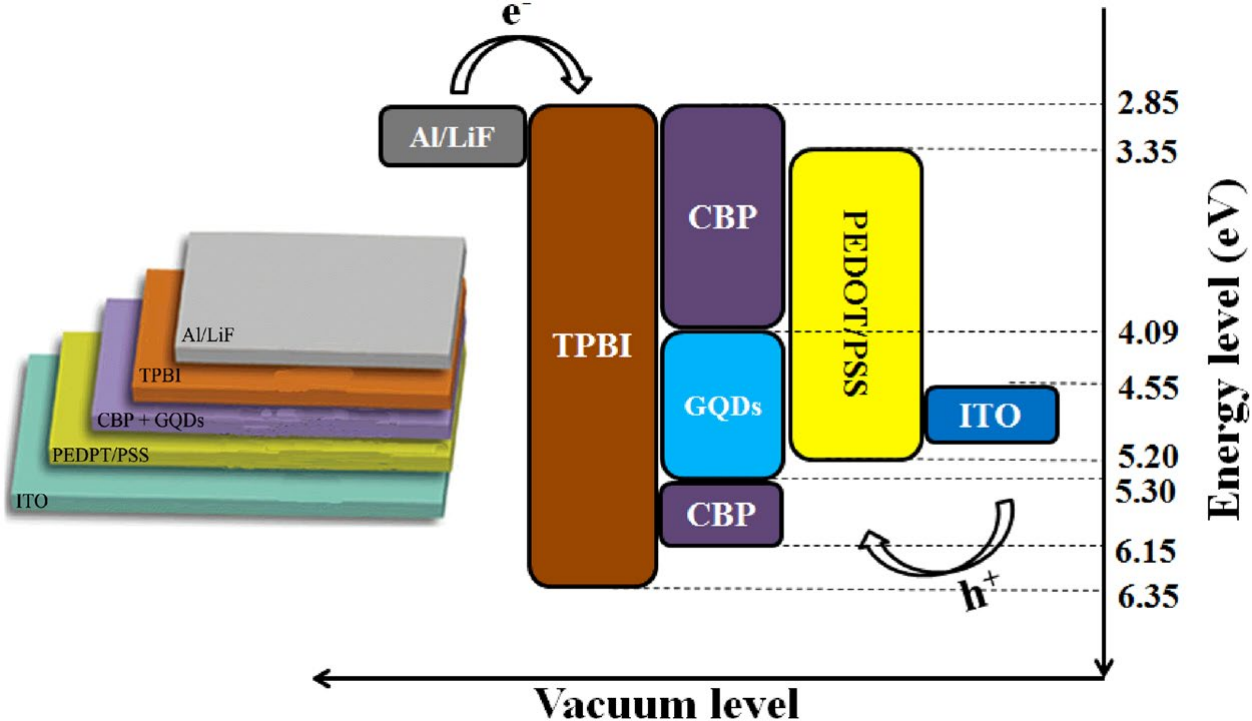
Energy level of the WLED device (using only GQDs as the white-light-emitting phosphor), the left inset is the schematic structure of the WLED device.

**Figure 8:**
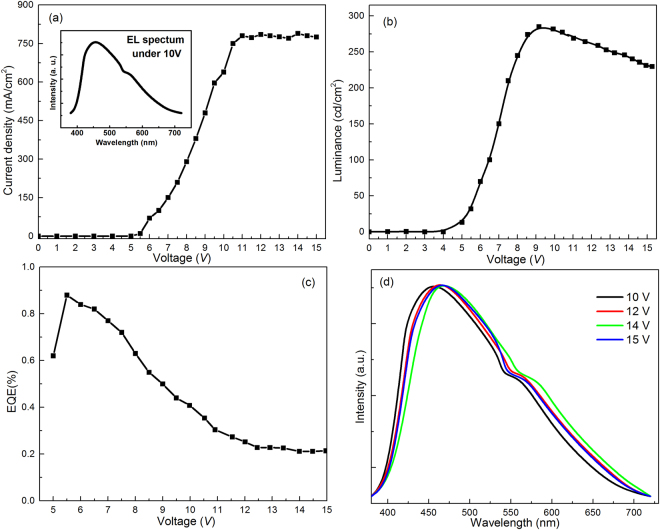
(**a**) *J-V* curve, the inset shows the EL spectrum, (**b**) Luminance of the WLED device, (**c**) EQE of WLED device and (**d**), EL spectra with different applied *V*.

For comparison, we first used solution-process to fabricate WLED by only using the GQDs(W) as white-light-emitting phosphor. The structure of the WLED device and the energy levels are shown in Fig. 7, which is mainly composed of patterned ITO anode, PEDOT: PSS hole injection layer, 40 nm GQDs doped CBP emitting layer, 40 nm TPBI electron transport layer, 0.8 nm LiF and 100 nm aluminum double layer as the cathode (Fig. 7). The energy transfer from CBP to GQDs is important for obtaining the WLED. The current density-voltage (*J-V*) curves are shown in the Fig. 8a, which indicate that the turn-on voltage is 6 V, the current density is stable at ~750 mA·cm^−2^ in the range between 10–15 V and the cold white light (450 nm + 560 nm EL of GQDs) is obtained when the voltage up to 10 V (inset of Fig. 8a). The highest luminance of WLED is ~280 cd·m^−2^ at the voltage of 11 eV (Fig. 8b), which is higher than the device from other fluorescent GQDs. EQEs of WLED for different applied voltages are 0.23% (6 V), 0.23% (9 V), 0.22% (11 V), 0.21% (13 V) and 0.19% (15 V) (Fig. 8c), which is all higher than other GQDs based LED devices, such as carbon dots^17^ or ZnO GQDs^36^. The EL spectra of the WLED under different *V* were shown in Fig. 8d. The Commission Internationale de l’Enclairage (CIE) coordinates are not located in the pure white light area (Supplementary Fig. S4), due to the lack of red component.

**Figure 9:**
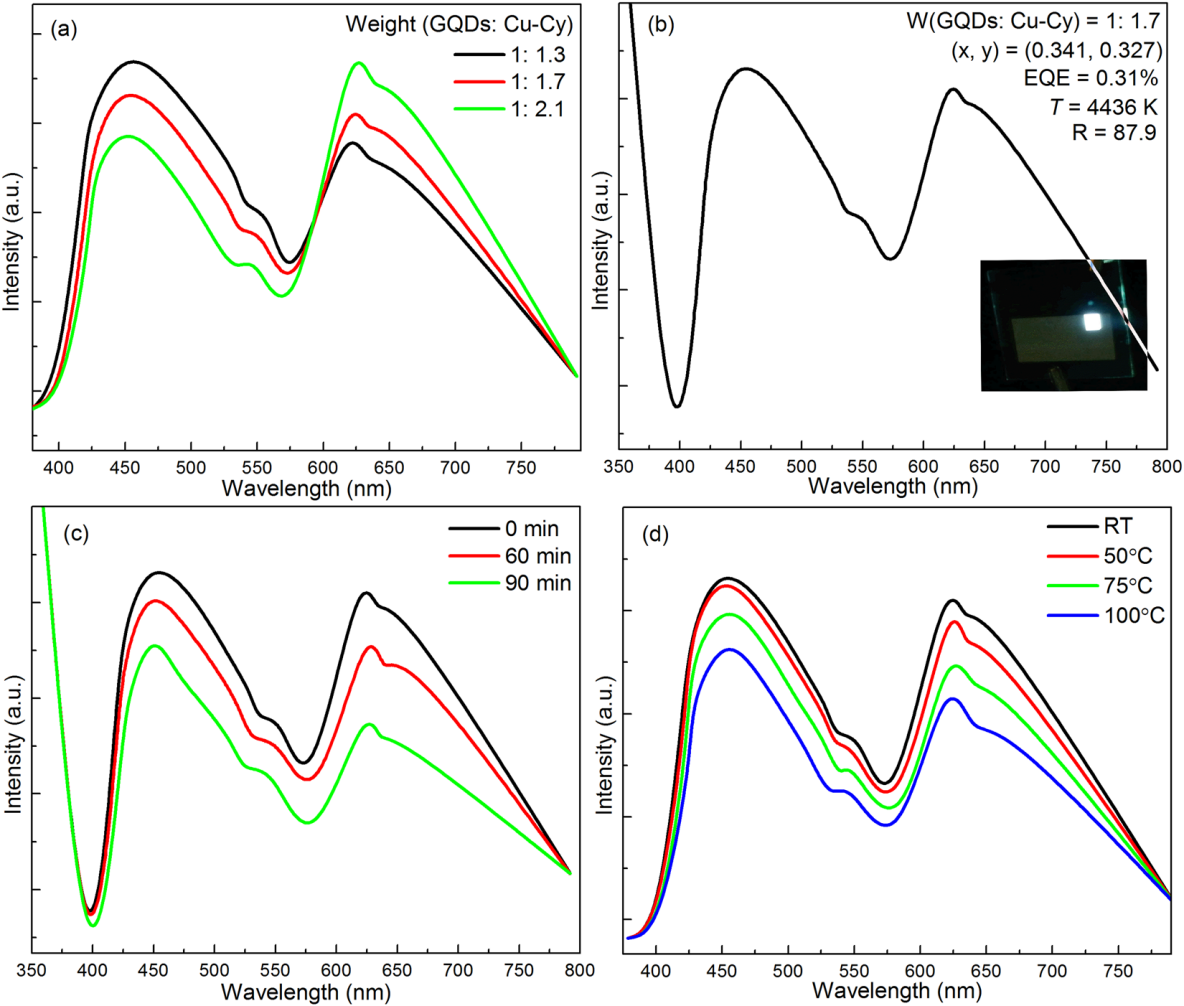
(**a**) PL spectra of phosphors with different weight rations between GQDs and Cu-Cy, (**b**) EL spectrum of the WLED at the forward bias current of 350 mA, (**c**) EL spectra for different operating times and (**d**), PL spectra at different temperature.

In order to solve the problem of lacking of red light, we added the Cu-Cy in the WLED structure. The emission spectra and CIE chromaticity positions of the mixed phosphors are shown in Fig. 9a, with the weight ratio of GQDs and Cu-Cy are 1: 1.3, 1: 1.7 and 1: 2.1, respectively. Under λ_ex_ = 365 nm, the emission bands are cover all the whole visible range from 380 to 780 nm, due to the additional bands originated from the emitting of Cu-Cy. Notably, the emission spectra of the materials are a mechanical mixture of their own emission spectrum from both the GQDs and Cu-Cy, which means that the emission intensity is enhanced with its ratio in the mixed compounds. From Fig. 9a, the color coordinates can be tuned by modifying the weight ratio of the two phosphors (Supplementary Fig. S5). The electroluminescence spectrum of the WLED device contains broad emissions (Fig. 9b and Supplementary Fig. S6, weight ratio at 1: 1.7, under forward bias current of 350 mA). The final performances of the device are: CIE (x, y) = (0.341, 0.327), *T* = 4436 K, *R* = 87.9 EQE = 0.31%, respectively. Furthermore, for discussing the stability of this device, Fig. 9c exhibits the emission of the WLED device at different operating times. Following time, both the two materials show decreasing of emission intensities and the red emission is decreased faster than the emission from the GQDs. In fact, following the operation times, the chip temperature would be higher than the RT, the shift of the self-activated luminescence with a large stokes (Cu-Cy) has a low thermal stability, thus the red emission is decreased faster. Additional, oxidation of the phosphors is another possible reason. To make it clear whether the intensity quenching is partially by oxidation, the device at different temperatures in air for half hour and then measured their luminescence spectra after cooling down to RT. As shown in Fig. 9d, the intensity decreases with the spectra shape remains unchanged. These phenomena indicate that partially of the samples were oxidized, and this oxidation is irreversible, which is the reason for the device to get worse following time.

Although this designed WLED device has a relatively good performance, it is sure that all of the properties of this system can be further improved by optimization, such as the ratio of epoxy resin to phosphor, the synthesis process for phosphors, LED chip fabrication process as well as employing the remote-phosphor LED configuration. Based on this study, it is convinced for us that enhanced white electroluminescence of WLED can be obtained *via* the RE free GQDs and Cu-Cy under single excitation as light emitting phosphors.

## Conclusions

In summary, we report new warm white color composites made of light emitting phosphors GQDs and Cu-Cy. Under a single wavelength excitation at λ_ex_ = 365 nm, these two materials show strong blue and red luminescence, respectively. GQDs with cold white fluorescence are synthesized *via* a facile microwave-assisted wet chemical reaction using graphite as the precursor. The structure and optical properties of the new red component phosphor were also investigated, which shows intense photoluminescence in the red range due to the transitions of the two different Cu^+^ ions in the composite. The warm white light emission with a quantum yield of 0.31% could be obtained by mixing these two self-activated luminescent materials at a weight ratio of 1: 1.7. Adopting a 365 nm near-UV chip for excitation, the LED composition feature good electroluminescent properties which exhibits an excellent color rendering index (87.9) and CIE coordinates (0.341, 0.327). These white light materials are environment friendly, easy to approach and cost-effective, which can potentially eliminate the challenge of RE resources. Moreover, a single chip is used for excitation instead of a multichip, which can further reduce the cost of the LED composition. We propose that this research could promote the practical application of GQDs/Cu-Cy in solid-state luminescence related fileds.

## Methods

### Preparation of GQDs

Mixed acid was prepared firstly by mixing the two acids in the volume ratio of H_2_SO_4_: HNO_3_ = 3: 1. 100 mg nanoscale graphite was then added into 85 ml of the mixed acid with ultrasonic shock for 3 h. Next, the dispersion was placed under the microwave irradiation for reacting (~3 h at 100 °C). Then, the resultant solution was filtered *via* the microporous membrane (pore size = 220 nm) and neutralized by Na_2_CO_3_. 320 ml deionized water (DW) was added into the neutralized solution for diluting. The GQDs aqueous solution was obtained by dialysis with dialysis bag (molecular weight cutoff = 1000). The pH of GQDs solution was regulated to be 12 and 30 ml of this GQDs solution was continued to react for 4 h at 200 °C under microwave irradiation. Finally, after further dialysis for 2 days, the GQDs aqueous solution was placed in refrigerator as raw material for preparing LED device.

### Synthesis of Cu-Cy particles

CuCl_2_·2H_2_O (0.92 g, 5.396 mmol) was dissolved in DW followed by addition of cysteamine (1.272 g, 16.488 mmol). Then, adding 5 M NaOH solution (16 ml) to adjust the pH value to 8. The mixed solution was stirred for 2 h at room temperature (RT) and then heated to its boiling temperature for 0.5 h. The Cu-Cy particles were gained by centrifuging and washing the crude product with DW and ethanol (at the volume ratio of DW: ethanol = 5: 4) three times followed by sufficient sonication. Finally, the particles were dried in a vacuum oven at 50 °C for 12 h.

### Fabrication of WLEDs

Firstly, the patterned ITO substrate was spin-coated by a layer of PEDOT:PSS with the thickness of 40 nm. Then, 4,4′-bis(carbazol-9-yl) biphenyl (CBP) in chlorobenzene (15 mg·ml^−1^) and GQDs (8 mg·ml^−1^) in toluene with the volume ratio of 9:1 were mixed together and formed the suspension. Next, the suspension contains CBP and GQDs was spin-coated on the layer of PEDOT:PSS, and then annealed at 80 °C for half hour to remove solvent for forming the emissive layer with the thickness of 40 nm. ITO coated with PEDOT: PSS, CBP and GQDs was transferred to a thermal evaporator chamber and then successively deposited with 1, 3,5-tri(phenyl-2-benzimidazolyl)-benzene (TPBI) (40 nm), LiF (0.8 nm) and Al (100 nm) under pressure of 5 × 10^−4^ Pa by thermal evaporation. The luminance of *J-V* characteristics of LED were detected using a Keithley source-meter (model 2602) combined with a calibrated luminance meter. Electroluminescence spectra were recorded by a HAAS-2000 system (Yuanfang, China). The thickness was measured by a spectroscopic ellipsometry (α-SE, Woollam Co. Inc.). All these processes were carried out at RT under ambient conditions. For comparison, another group of WLEDs were also fabricated by co-depositing the GQDs and Cu-Cy with various ratio (weight) on the same NUV LED chip (365 nm). The optical and electroluminescence properties were measured in the same way.

### Characterizations

X-ray diffraction (XRD) was performed *via* a Bruker D8-Advance X-ray diffractometer with Cu Ka radiation (λ = 0.15405 nm). The photoluminescence and photoluminescence excitation (PL and PLE) spectra were recorded on an F4500 fluorescence spectrometer. The luminescence quantum yields and fluorescent lifetimes were measured by an FLS920 spectrofluorometer (Edinburgh Inc.) equipped with integrating sphere. Morphology was observed on a field emission scanning electron microscope (FE-SEM, S-4800 Hitachi) operating at 15 kV and a transmission electron microscope (TEM, JEOL JEM 2100 F) operating at 200 kV. The temperature quenching on the luminescent properties of the WLEDs was studied by heat treating the composite at different temperatures in the air for half hour. Raman spectra were measured by Renishaw InVia microscope with 532 nm laser beam as the excitation source. FTIR spectra were measured with Shimadzu IRPrestige-21FTIR spectrophotometer. XPS investigation was conducted using PHI 5000 VersaProbe system with Al cathode as the X-ray source. Height images of nanoscale samples were evaluated by the atomic force microscopy (AFM, Oxford Inc. MFP-3D Infinity).

### Calculations

Adopting First-principle for calculation the band structure and density of states (DOS) of Cu-Cy, hybird density functional theory (DFT) was performed *via* a single point with the generalized gradient approximation (GGA) lattice geometry^37^. The GGA scheme was firstly employed for the fully relaxed structural optimization. Next, the screened-exchange hybrid functional of Heyd, Scuseria and Ernzerhof (HSE) representing the electronic exchange-correlation energy was adopted during calculation of the DOS and band structure^38^. According to the projected augmented wave (PAW) method, the valence configurations (valence and semi-core electrons) of the 1s^2^, 2s^2^2p^2^, 2s^2^2p^3^, 3s^2^3p^2^, 3s^2^3p^5^ and 4s^1^3d^10^ for the elements H, C, N, S, Cl and Cu were obtained. The model structure is selected a 96-atom conventional cell. The cutoff energy was 450 eV and the Monkhorst-Pack K-point mesh for the unit cell was 3 × 1 × 3. When the total energy was converged to 10^−5^ eV and the residual forces on atoms were below 0.02 eV·Å^−1^, the structural relaxation of the crystal was ended.

### Data availability Statement

The datasets generated during and/or analyzed during the current study are available from the corresponding author on reasonable request.

## Electronic supplementary material

Supplementary information
