# Supplementary material for: Rare-Earth Free Self-Activated Graphene Quantum Dots and Copper-Cysteamine Phosphors for Enhanced White Light-Emitting-Diodes under Single Excitation
Source: Sci Rep. 2017 Oct 9;7:12872. doi: 10.1038/s41598-017-13404-1 (PMC5634462; doi:10.1038/s41598-017-13404-1)
Supplement: Supplementary file 1 — Supplementary information [file 41598_2017_13404_MOESM1_ESM.doc]

Supplementary Information for

Rare-Earth Free Self-Activated Graphene Quantum Dots and Copper-Cysteamine Phosphors for Enhanced White Light-Emitting-Diodes under Single Excitation

Wubin Dai ^1,*^, Yifeng Lei ^2^, Man Xu ^1^, Pei Zhao ^1^, Zhanhui Zhang ^1^ and Jia Zhou ^1^

^1^ Key Laboratory for Green Chemical Process of Ministry of Education, Hubei Key Laboratory of Plasma Chemistry and Advanced Materials, School of Material Science and Engineering, Wuhan Institute of Technology, Wuhan 430205, Hubei, China

^2^ School of Power and Mechanical Engineering, Wuhan University, Wuhan 430072, Hubei, China

^*^ Corresponding author, E-mail: [wubin.dai@foxmail.com](mailto:wubin.dai@foxmail.com)


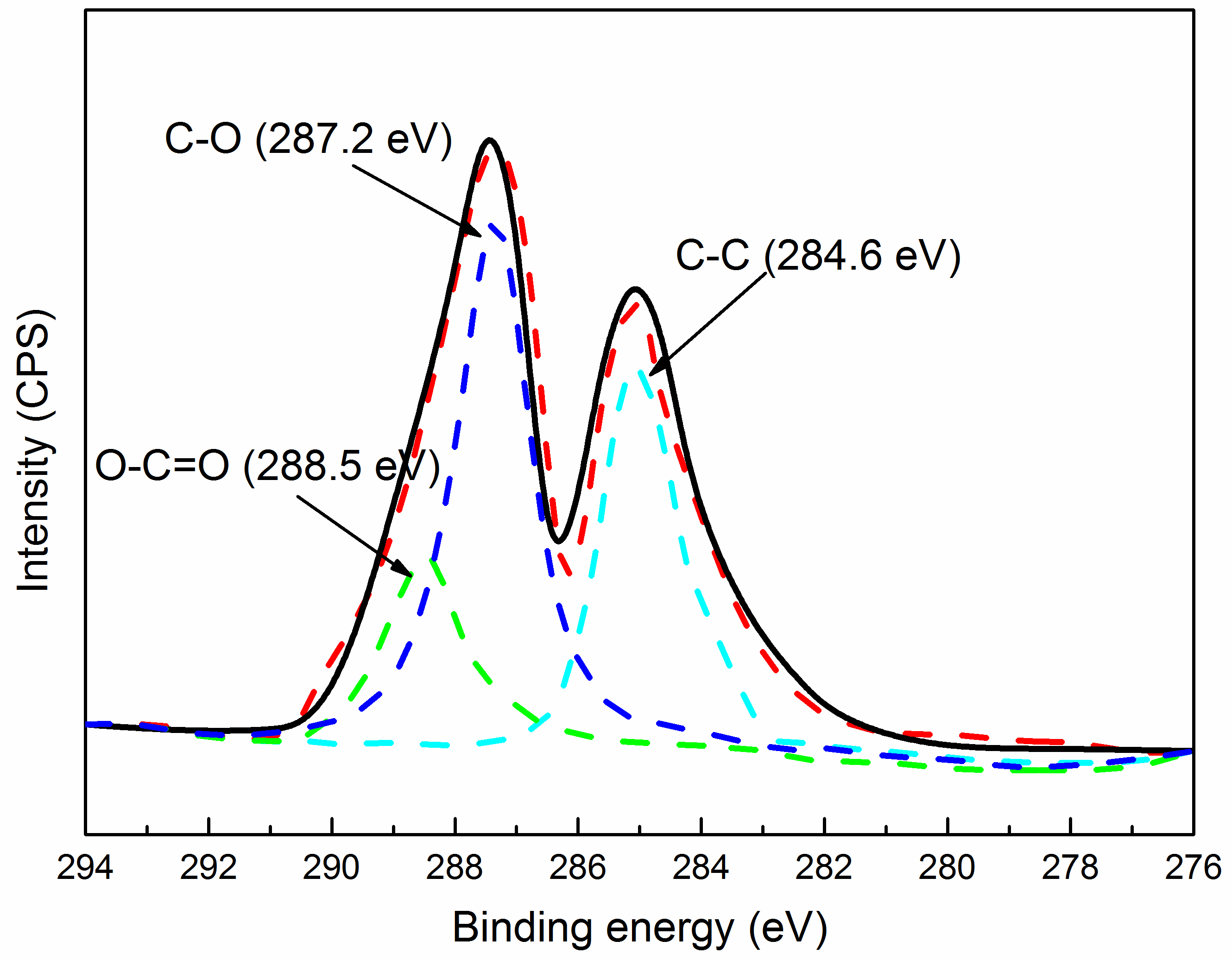


**Figure S1.** High-resolution C1s XPS spectrum of GQDs(Y).

**
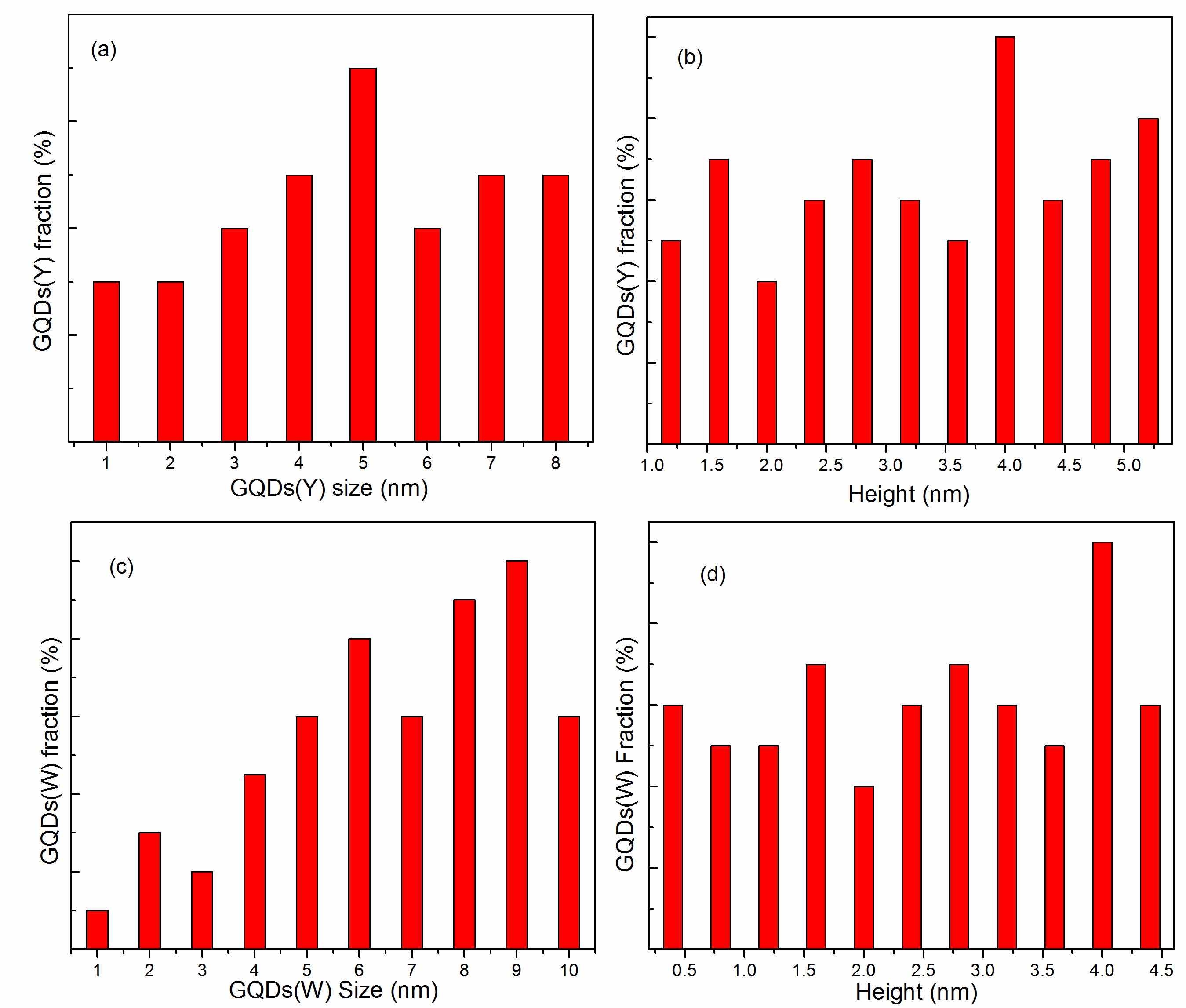
**

**Figure S2.** Particle size distribution of GQDs(Y) (a) and GQDs(W) (c), height distribution of GQDs(Y) (b) and GQDs(W) (d).


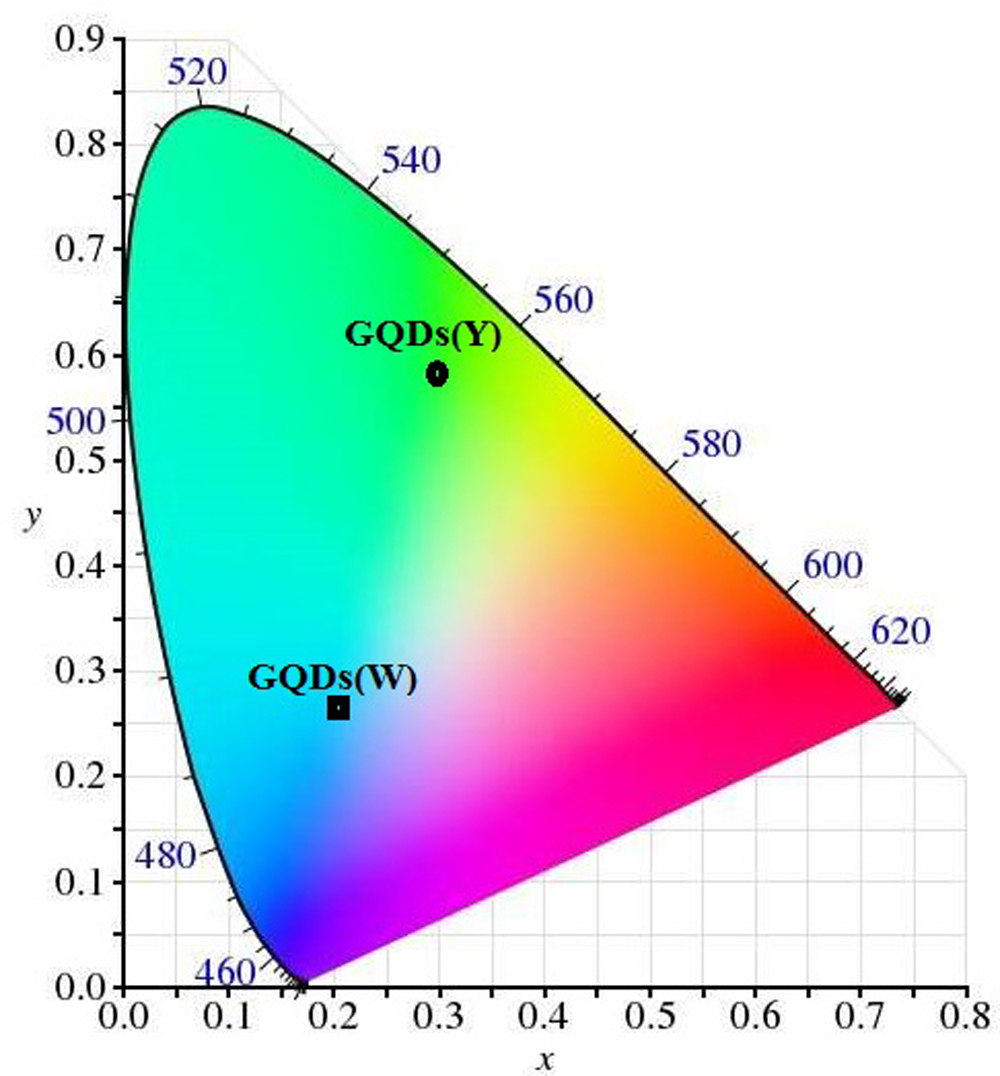


**Figure S3.** CIE coordinates of the PL spectra GQDs(Y) (0.346, 0.587) and GQDs(W) (0.226, 0.261).

**
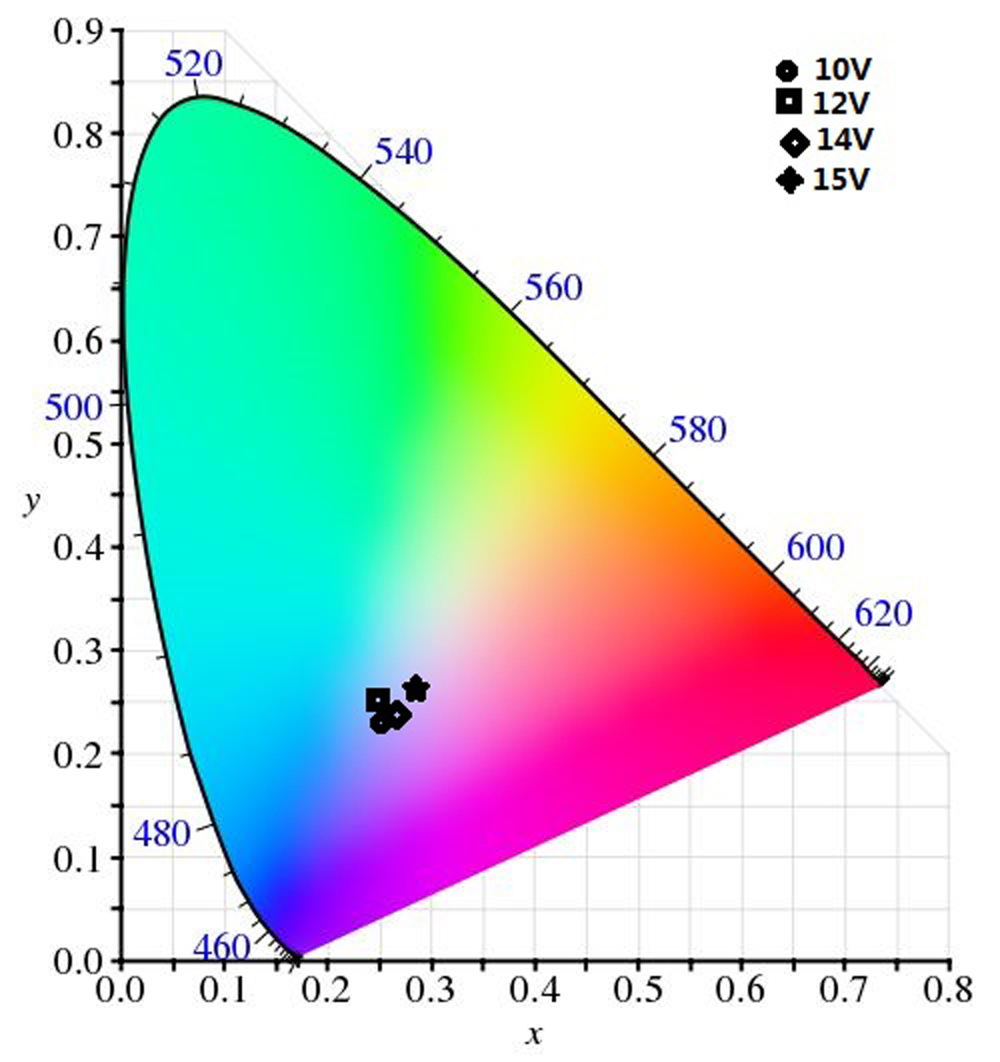
**

**Figure S4.** Commission Internationale de l’Enclairage (CIE) coordinates for the samples applied in different *V* (PL spectra).


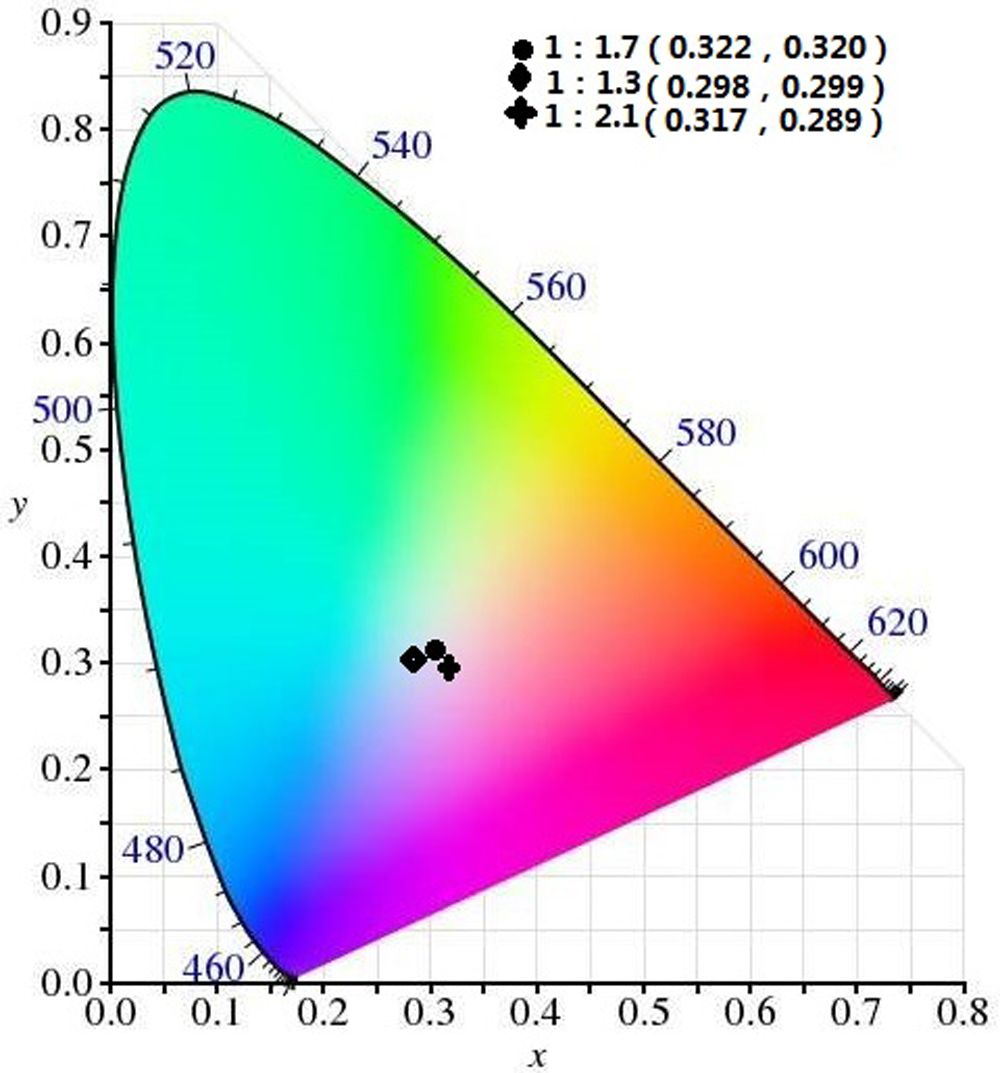


**Figure S5.** Commission Internationale de l’Enclairage (CIE) coordinates for the samples with different weight ratio of GQDs(W) and Cu-Cy (PL spectra).


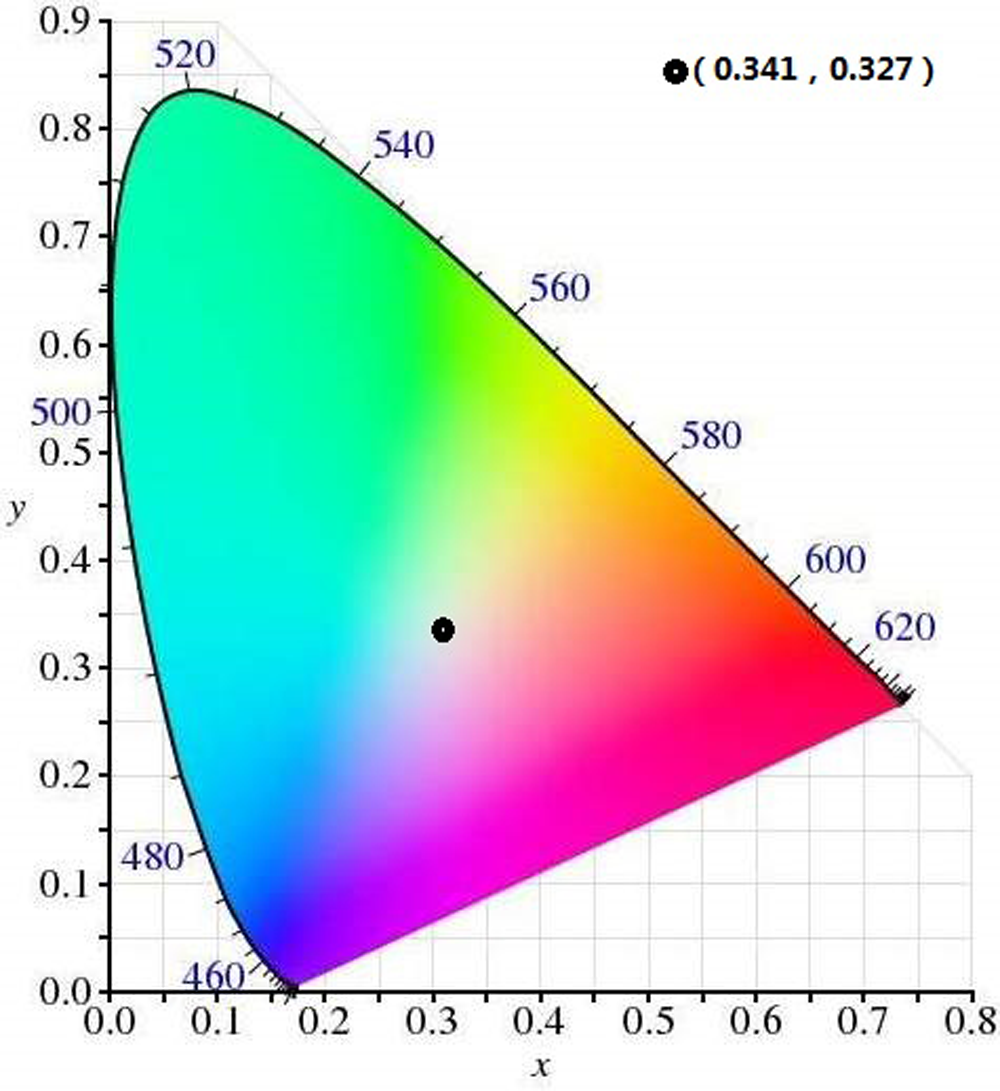


**Figure S6.** Commission Internationale de l’Enclairage (CIE) coordinates for the sample with weight ratio of GQDs(W) and Cu-Cy: 1: 1.7 (EL spectrum).
